# Erectile and sexual dysfunction in male and female patients with celiac disease: A cross‐sectional observational study

**DOI:** 10.1111/andr.13186

**Published:** 2022-04-26

**Authors:** Lorenzo Romano, Raffaele Pellegrino, Carmine Sciorio, Biagio Barone, Antonietta Gerarda Gravina, Antonio Santonastaso, Caterina Mucherino, Silvia Astretto, Luigi Napolitano, Achille Aveta, Savio Domenico Pandolfo, Davide Loizzo, Francesco Del Giudice, Matteo Ferro, Ciro Imbimbo, Marco Romano, Felice Crocetto

**Affiliations:** ^1^ Department of Neurosciences Reproductive Sciences and Odontostomatology University of Naples “Federico II” Naples Italy; ^2^ Hepato‐Gastroenterology Unit Department of Precision Medicine University of Campania “Luigi Vanvitelli” Naples Italy; ^3^ Urology Unit ASST “Alessandro Manzoni” Hospital Lecco Italy; ^4^ Gastroenterology Unit “Sant'Anna and San Sebastiano” Hospital Caserta Italy; ^5^ Division of Urology Department of Surgery VCU Health Richmond Virginia USA; ^6^ Urology Andrology and Kidney Transplantation Unit Department of Emergency and Organ Transplantation University of Bari “Aldo Moro” Bari Italy; ^7^ Department of Maternal‐Infant and Urological Sciences Policlinico “Umberto I” Hospital University of Rome “La Sapienza” Rome Italy; ^8^ Department of Urology Stanford Medical Center Stanford California USA; ^9^ Division of Urology European Institute of Oncology IRCSS Milan Italy

**Keywords:** celiac disease, FSFI, IIEF‐5, sexual dysfunction

## Abstract

**Introduction:**

Sexual function is often impaired in patients with chronic illnesses. Several patients with chronic gastrointestinal and liver disorders have been shown to suffer from sexual dysfunction, and celiac disease is a highly prevalent gastroenterological disorder.

**Aim:**

The aim of this study was to investigate the sexual function incidence and the risk factors for sexual dysfunction in both male and female celiac disease patients.

**Methods:**

Two hundred and eighty‐four patients (170 females, 114 males) participated in this cross‐sectional observational study in an anonymous manner. Female sexual function was assessed through the Female Sexual Function Index questionnaire. Male sexual function was assessed through the International Index of Erectile Function‐5 questionnaire. Clinical‐demographic variables were recorded. We investigated differences in the patient‐reported outcomes among the different subgroups and whether there were clinical‐demographic predictors of sexual dysfunction in our setting.

**Main outcome measures:**

Prevalence and assessment of sexual dysfunction in celiac disease patients.

**Results:**

In the female group, 85 subjects (50%) had a total score compatible with sexual dysfunction: 43 (61.42%) showed low desire, 79 (46.47%) showed arousal disorder, 66 (38.82%) lubrication disorder, and 84 (49.41%) inability of obtaining an orgasm. Also, a large proportion of our female patients, 161 (94.70%), showed sexual discomfort during intercourse. In the male group, 79 patients (62.2%) showed scores compatible with normal erectile function, eight (7.01%) had mild erectile dysfunction, 24 (21.05%) mild to moderate erectile dysfunction, and three (2.63%) presented severe erectile dysfunction. Altered body mass index was significantly associated with sexual dysfunction both in male and female patients. Early age at diagnosis was a significant predictor of sexual dysfunction in male celiac disease patients.

**Conclusions:**

A significant proportion of celiac disease patients present sexual dysfunction. Early age at diagnosis and high body mass index seem to predict sexual dysfunction in this clinical setting. Assessment of sexual function should be part of the initial evaluation of celiac disease patients in order to establish a prompt diagnosis and early treatment.

## 1 INTRODUCTION

Sexual dysfunctions (SD) are the most prevalent psychological disorder in the general population. The prevalence of SD in women varies from 40% to 50% irrespective of age.^1^ In contrast prevalence of erectile dysfunction (ED) in men largely depends on age, ranging from 1% to 10% in those younger than 40 years, from 2% to 15% in those from 40 to 49 years, and from 20% to 40% in those between 60 and 69 years.^1^ Mounting evidence indicates that a number of patients with chronic gastrointestinal and hepatic disorders may suffer from SD with a significant alteration of their quality of life.^2^ According to the Diagnostic and Statistical Manual of Mental Disorder (DSM‐V), SD are classified into four major categories which include, overall, disorders of sexual desire, arousal, orgasm, and sexual pain.^3^ In males, the most prevalent disorder is related to ED followed by premature ejaculation, whereas in women dyspareunia, that is, pain during sexual intercourse, and hypoactive desire represent the most common sexual disorders.^4^, ^5^ The etiopathogenesis of SD is widely multifactorial and includes physiological, anatomical, and socio‐cultural differences.^6^ The pathogenesis of SD in chronic gastrointestinal disorders is multifactorial and may be contributed to by psychological problems, chronic inflammation with endothelial dysfunction, endocrine disturbance, or drug‐related factors.^7^ Evidence shows that untreated celiac disease (CD) patients had a significantly lower frequency of intercourse and an overall lower satisfaction regarding their sexual life, which drastically improve after gluten‐free diet.^8^

However, there are no studies that have systematically evaluated sexual function in CD patients using well‐established questionnaires. Therefore, this observational cross‐sectional study was designed to assess sexual function in CD patients through validated questionnaires exploring female and male sexual activity. As secondary endpoints, the role of a number of clinical‐demographic variables in the sexual function score of CD patients was established.

## 2 METHODS

### 2.1 Study design and population

We conducted a cross‐sectional observational study at the outpatient clinics for CD of the Hepato‐Gastroenterology Unit of the University Hospital of University of Campania “Luigi Vanvitelli” and of the Gastroenterology and Digestive Endoscopy Unit of the “Sant'Anna and San Sebastiano” General Hospital, Caserta, Italy.

Patient enrollment took place from March to December 2021.

Those meeting the following inclusion criteria were considered eligible for the study: age greater than 18 years, and well‐established diagnosis of CD (i.e., elevated serum levels of anti‐transglutaminase IgA antibodies plus histology). All patients were on a gluten‐free diet. We excluded from the study: patients with legal inability to provide free consent for participation, patients with established psychiatric disorders, patients with previously established sexual sphere disorders and, as a consequence, those using 5‐phosphodiesterase inhibitors, and patients with clinically significant infections within 6 months of enrollment, as well as patients with neoplasia.

The following variables were collected: sex, age, age at diagnosis of CD, job, weight (in kilograms), height (in centimeters), body mass index (BMI), how long the patient had been following a gluten‐free diet (in months), smoking status, alcohol consumption, total number of sexual partners at the time of enrollment, others comorbidities, medications taken (with particular attention to the possible intake of antidepressants or anxiolytics), and main CD‐associated symptom/signs complained by the patient.

With regard to the smoking status, we have considered both the current and previous status of smoker. With regard to the use of alcohol, we considered, as non‐consumer, patients who totally denied the use of alcohol, occasional users who consumed no more than two alcohol units (i.e., 1 alcohol unit = 12 g of ethanol) per week, and frequent users who consumed more than two alcohol units per week.

All CD patients enrolled in the study were given an anonymous questionnaire, according to gender, considering stratifying gender into males and females.

The primary endpoint of the study was to estimate the levels of female and male SD in our sample of CD patients. The secondary endpoint was to study how the clinical‐demographic characteristics of patients related to the scores of the validated questionnaires used. This study was conducted according to the principles of World Medical Association Declaration of Helsinki and informed consent from patients was obtained. The ethics committee of our department approved the study.

### 2.2 Sexual dysfunction assessment in female patients

Female patients were given the Female Sexual Function Index (FSFI) questionnaire, a widely validated questionnaire used in clinical practice.^9^, ^10^ This questionnaire consists of 19 questions, each being part of a domain. It explores sexual desire (questions 1 and 2), sexual arousal (questions 3–6), lubrication (questions 7–10), orgasm (questions 11–13), sexual satisfaction (questions 14–16), and pain associated with sexual activity (questions 17–19). Each domain has a minimum score (0 or 1.2) to a maximum score of 6. Each domain score is multiplied by a multiplier, resulting in a domain subscore. All subscores are summed to obtain the final score. The lower the total score, therefore, the less the impairment of female sexual function. The cutoff considered in order to detect female SD was ≤23.45.^11^

### 2.3 Erectile dysfunction assessment in male patients

Male patients were administered the International Index of Erectile Function‐5 (IIEF‐5) questionnaire.^12^, ^13^ The questionnaire explores five domains (A–E) each with six scored responses ranging from 0 to 5: the ability to achieve and maintain a penile erection (A), achieving a penile erection sufficient for penetration (B), maintaining a penile erection after penetration (C), difficulty in maintaining a penile erection until the end of sexual activity (D), and finally pleasure during sexual activity (E). A total score between 22 and 25 is compatible with normal sexual penile function. A score between 17 and 21 indicates mild ED, between 12 and 16 indicates moderate ED, and between 5 and 7 indicates severe ED.^14^

### 2.4 Analysis

Continuous variables were expressed as median (interquartile range) and ordinal variables, expressed as numerosity (percentage of total).

We proceeded to check the normality of distribution of the variables via the Kolmogorov–Smirnov test. Spearman's rho test was used to evaluate the correlation between two variables. The Mann–Whitney *U*‐test or, alternatively, the Kruskal–Wallis test, was used to evaluate whether the distribution of a variable was statistically different between the groups. Multivariate analysis models by logistic regression were used to identify predictors of SD. Data were expressed as odds ratios (ORs) with relative 95% confidence interval (CI) and relative *p*‐value. A two‐tailed *p*‐value <0.05 was considered statistically significant. The relative 95% CI was expressed. IBM SPSS was used to perform the statistical analyses. Prism was used for graphing.

## 3 RESULTS

### 3.1 Baseline characteristics

The baseline characteristics of our cohort of 170 females (59.9%) and 114 males (40.1%) patients are illustrated in Table 1.

**TABLE 1:** Baseline characteristics of the study population

Subgroups	Females (*N* = 170), median (IQR) or *N* (%)	Males (*N* = 114), median (IQR) or *N* (%)	*p*‐Value^a^ (95% CI)
Age (years)	32 (25–42.5)	33 (24–46.25)	0.760 (0.752–0.769)
Age at diagnosis (years)	30 (21.75–39)	31 (19–43)	0.741 (0.732–0.749)
Weight (kg)	58 (53–67)	83 (75.5–88)	**0.001** (0.0001–0.002)
Height (cm)	166 (160–170)	179 (176–186)	**0.001** (0.0001–0.002)
BMI	21.8 (20.2–24.1)	25.2 (23.7–28.7)	**0.001** (0.0001–0.002)
Gluten‐free diet (months)	36 (18–87)	61 (23.7–28.7)	**0.001** (0.0002–0.001)
Smoking status			
Yes	49 (28.8%)	30 (26.3%)	0.205 (0.197–0.213)
No	101 (59.4%)	53 (46.5%)	
Past smoker	20 (11.8%)	31 (27.2%)	
Alcohol use			
Non‐consumer	61 (35.9%)	30 (26.3%)	**0.031** (0.027–0.034)
Occasionally	105 (61.8%)	84 (73.7%)	
Frequently	4 (2.4%)	–	
Number of total sexual partners			
0	20 (11.8%)	–	**0.001** (0.0001–0.002)
1	65 (38.2%)	32 (28.1%)	
2	26 (15.3%)	23 (20.2%)	
3	28 (16.5%)	6 (5.3%)	
4	9 (5.3%)	7 (6.1%)	
5	8 (4.7%)	–	
>5	14 (8.2%)	46 (40.4%)	
Comorbidity			
Sideropenic anemia	14 (8.2%)	–	0.143 (0.136–0.150)
Hashimoto's thyroiditis	11 (6.5%)	–	
Hypertension	7 (4.1%)	8 (7%)	
Diabetes mellitus	9 (5.3%)	11 (9.6%)	
Rheumatoid arthritis	2 (1.2%)	–	
Bronchial asthma	2 (1.2%)	20 (17.5%)	
Bronchiectasis	2 (1.2%)	–	
Atopic dermatitis	2 (1.2%)	–	
Seborrheic dermatitis	2 (1.2%)	–	
Migraine	2 (1.2%)	–	
Fibromyalgia	2 (1.2%)	–	
Irritable bowel syndrome	2 (1.2%)	–	
Peripheral venous insufficiency	–	4 (3.5%)	
Medication			
Iron supplements	16 (9.4%)	8 (7%)	**0.001** (0.0009–0.002)
Vitamin D supplements	6 (3.5%)	–	
Levothyroxine	19 (11.2%)	–	
Birth‐control pill	6 (3.5%)	–	
Triptan	2 (1.2%)	–	
Acetaminophen	4 (2.4%)	10 (8.8%)	
Antihistamines	1 (0.6%)	8 (7%)	
Antidepressants	5 (2.9%)	–	
Anxiolytics	5 (2.9%)	–	
ACE inhibitors	–	7 (6.1%)	
Steroids	–	12 (10.5%)	
Ibuprofen	–	7 (6.1%)	
Insulin	–	7 (6.1%)	
Sulodexide	–	4 (3.5%)	

Abbreviations: ACE, angiotensin‐converting enzyme; BMI, body mass index; CI, confidence interval; IQR, interquartile range.

^a^ *p*‐Value indicates whether the distribution of variables was statistically different between males and females. Significant *p*‐values are highlighted in bold.

The overall median age was 32 (25–44) years. Patients had been diagnosed with CD at a median age of 31 (20–40) years and had been on a gluten‐free diet for 40.5 (23.25–85) months. The overall BMI value was 23.73 (20.68–25.61).

In the female population, we observed that most patients did not report comorbidities (122, 71.77%) while the minority (48, 28.23%) reported at least one comorbidity. Overall, 64 (37.65%) females were on medication. In the male population, most patients (71, 62.28%) had no comorbidities while the minority (43, 37.72%) reported to have at least one condition.

Sixty‐three (55.1%) of the male patients were taking medications. None was using anxiolytics or antidepressants. Other clinical‐demographic features are shown in Tables 2 and 3.

**TABLE 2:** Female Sexual Function Index (FSFI) scores distribution concerning different clinical‐demographic variables

Subgroup	*N*	%	Total FSFI score, median (IQR)	*p*‐Value^a^ (95% CI)
Total sample	170	100%	23.55 (9.8–25.2)	**–**
Main symptom				
Anemia	22	12.9%	24.2 (24.2–28.2)	**0.0001 (0.00001– 0.0003)**
Dermatitis	13	7.6%	3.8 (3.8–25.2)	
Diarrhea	30	17.6%	22.8 (15.9–24.1)	
Abdominal pain	28	16.4%	23.7 (11.27–26.4)	
Bloating	33	19.4%	23.4 (5.4–29.8)	
Nausea	6	3.5%	7.85 (6.3–9.4)	
Weight loss	4	2.3%	2 (2–2)	
Asthenia	16	9.4%	17.1 (9.8–27.77)	
Asymptomatic	18	10.5%	23.9 (16.6–26.4)	
Job				
Unemployed	44	25.8%	27.9 (22.8–29.8)	**0.0001 (0.00001–0.0003)**
Employed	60	35.2%	24.2 (9.4–25.2)	
Self‐employed	12	7.05%	16.9 (15.7–21.8)	
Freelancer	25	14.7%	15.9 (9.8–23.4)	
Student	29	17%	6.3 (3.8–25)	
Partner number				
0	20	11.76%	3.8 (3.8–4.4)	**0.0001 (0.00001–0.0003)**
1	65	38.2%	23.9 (15.9–27.9)	
2–5	71	41.7%	24.1 (18.5–25.2)	
>5	14	8.2%	16.9 (9.4–30.4)	
Smoking status				
No	101	59.4%	25.8 (15.8–27.6)	**0.01 (0.008–0.012)**
Yes	49	28.8%	24.2 (3.8–25)	
Past smoker	20	11.7%	9.8 (9.8–16.9)	
Alcohol use				
Non‐consumer	61	35.8%	24.2 (21.2–27.6)	0.103 (0.097–0.109)
Occasionally	105	61.7%	18.5 (7.9–25.2)	
Frequently	4	2.3%	23.4 (23.4–23.4)	

Abbreviations: CI, confidence interval; IQR, interquartile range.

^a^ *p*‐Value indicates significance of differences in FSFI scores between different subgroups. Significant *p*‐values (*p* < 0.05) are highlighted in bold.

**TABLE 3:** International Index of Erectile Function questionnaire‐5 (IIEF‐5) scores distribution in relation to different clinical‐demographic variables

Subgroup	*N*	%	Total IIEF‐5 score, median (IQR)	*p*‐Value^a^ (95% CI)
All males	114	100%	23 (20–25)	–
Main symptom				
Anemia	18	15.7%	15.7%	0.174 (0.167–0.182)
Dermatitis	7	6.1%	6.1%	
Diarrhea	55	48.2%	48.2%	
Abdominal pain	6	5.2%	5.2%	
Bloating	6	5.2%	5.2%	
Nausea	4	3.5%	3.5%	
Weight loss	–	–	–	
Asthenia	–	–	–	
Asymptomatic	18	15.7%	15.7%	
Job				
Unemployed	7	6.1%	24 (22–25)	**0.0001 (0.0001–0.0002)**
Employed	38	33.3%	22 (15–24)	
Self‐employed	7	6.1%	20 (20–20)	
Freelancer	36	31.5%	23 (14–23)	
Student	26	22.8%	24.5 (23.75–25)	
Partner number				
0	–	–	–	**0.008 (0.007–0.010)**
1	32	28%	24 (14–24)	
2–5	36	31.5%	22.5 (15–25)	
>5	46	40.3%	23 (22–25)	
Smoking status				
Yes	30	26.3%	23 (14–24.25)	0.526 (0.517–0.536)
No	53	46.4%	24 (15–25)	
Past smoker	31	27.1%	23 (20–23)	
Alcohol use				
Non‐consumer	28	24.5%	20 (14–23.75)	**0.005 (0.003–0.006)**
Occasionally	78	68.4%	23 (22–25)	
Frequently	8	7%	23.5 (16.75–25)	

Abbreviations: CI, confidence interval; IQR, interquartile range.

^a^ *p*‐Value indicates significance of differences in IIEF‐5 scores between different subgroups. Significant *p*‐values (*p* < 0.05) are highlighted in bold.

### 3.2 Sexual function in female patients

The median FSFI score in our population of 170 female patients with CD was 23.5 (9.8–25.2) (Table 2). We observed that 85 female patients (50%) had a total score compatible with some degree of SD: 43 (61.42%) showed low desire, 79 (46.47%) showed arousal disorder, 66 (38.82%) lubrication disorder, 84 (49.41%) orgasm disorder while 161 (94.70%), finally, showed a score suggestive of sexual pain.

Total FSFI score largely depended on the type of symptoms reported by the patient, the type of work, number of total sexual partners, and smoking status (Table 2).

We investigated which continuous clinical‐demographic variables correlated significantly with total FSFI score. We found significant correlation with weight (*p* < 0.001, *ρ* = 0.270), height (*p* = 0.003, *ρ* = ‐ 0.228), and consequently BMI (*p* < 0.001, *ρ* = 0.431).

Moreover, operating a stratification between women with compromised FSFI score and women with normal FSFI score, we did not observe differences regarding median age at diagnosis of CD, which was 34 (21.5–40) in the first group and 26 (21.5–32.5) in the second group (*p* = 0.057) or median number of months on gluten‐free diet, which were 36 (16–84) months and 28 (18–96) months, respectively (*p* = 0.395).

Regarding comorbidities, we found that in 14 (8.2%) patients with sideropenic anemia, median FSFI value was 17.1 (9.4–21.8). Also, in 11 (6.5%) patients with Hashimoto's thyroiditis had a significant lower FSFI score [9.9 (9.8–25.2) (*p* < 0.001)]. Conversely, arterial hypertension and diabetes did not seem to have a statistically significant influence on FSFI scores (*p* = 0.601; 0.336).

Use of antianxiety drugs (i.e., benzodiazepines) did not significantly affect FSFI scores. In five patients (2.9%) who reported using benzodiazepines, FSFI score was not significantly different than observed in non‐benzodiazepine users, with a median of 28.2 (15–28.2) versus 23.4 (9.8–25.1), respectively (*p* = 0.196).

As a result, at multivariate analysis, we observed that a higher BMI value (*p* = 0.007; OR 0.859, 95% CI 0.770–0.959) was shown to behave as a predictor of poor female sexual function.

In contrast, age (*p* = 0.389; OR 0.947, 95% CI 0.838–1.071), age at diagnosis (*p* = 0.150; OR 1.097, 95% CI 0.967–1.245), number of months on a gluten‐free diet (*p* = 0.461; OR 1.005, 95% CI 0.992–1.017), presence of hypertension (*p* = 0.688; OR 0.683, 95% CI 0.107–4.439), and presence of diabetes (*p* = 0.281; OR 2.563, 95% CI 0.463–1.418) were not predictors.

We also assessed whether the serum level of anti‐transglutaminase antibodies had any influence on FSFI score and found no correlation (data not shown).

### 3.3 Sexual function in male patients

In our sample of 114 male participants, the general male sexual function median score, assessed as IIEF‐5 score, was 23 (20–25) (Table 3). In detail, 79 patients (69.3%) showed scores compatible with normal erectile function, eight (7.01%) males had mild ED, 24 (21.05%) mild to moderate ED, and three (2.63%) presented IIEF‐5 scores compatible with severe ED.

We observed a significant correlation between IIEF‐5 score compatible with SD and increasing age (*p* = 0.019, *ρ* = = ‐0.220), early age at diagnosis of CD (*p* = 0.029, *ρ* = ‐0.204), and high BMI (*p* < 0.001, *ρ* = ‐0.334).

We found that pleasure‐related scores during sexual activity were significantly higher in nonsmokers or ex‐smokers compared with smokers (*p* = 0.001).

The majority of our patients (107, 93.8%), who had no hypertension, had a median IIEF‐5 score of 23 (20–25), compared with patients (seven, 6.2%) with hypertension, who showed significantly lower IIEF‐5 score, with a median of 15 (15–15) (*p* = 0.045).

As shown in Table 3, patients whose main CD‐related complaint was bloating, showed worse sexual function related to obtaining an erection sufficient for initiating and/or maintaining intercourse and experienced less pleasure (*p* < 0.05). Job also seemed to affect sexual function in our clinical setting.

**FIGURE 1:**
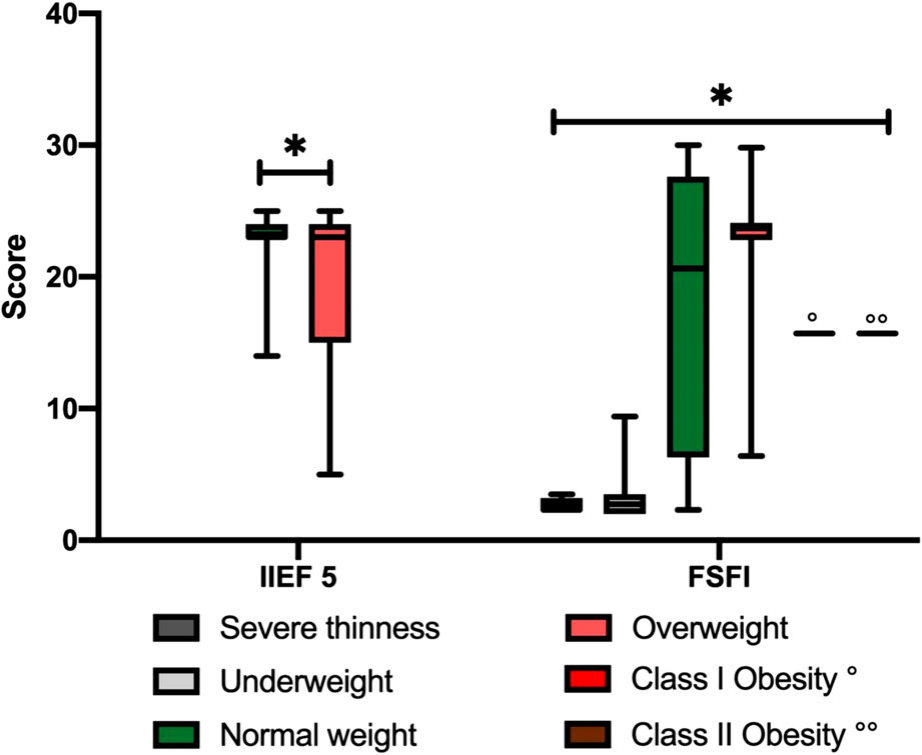
Changes in male (International Index of Erectile Function‐5 [IIEF‐5]) and female (Female Sexual Function Index [FSFI]) sexual function in relation to body mass index (BMI) classes (severe thinness <16.5, underweight 16.5–18.49, normal weight 18.5–24.99, overweight 25–29.99, class I obesity 30–34.99, class II obesity 35–39.99). It can be observed that in both males and females, as BMI worsens, sexual function also worsens significantly (*p < 0.05). Data are expressed as median and relative range

**FIGURE 2:**
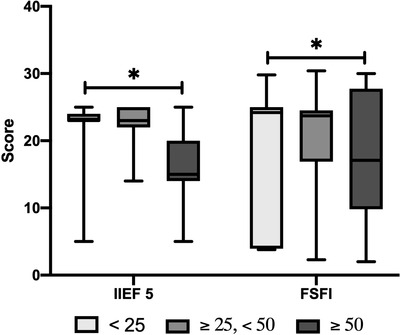
Changes in male (International Index of Erectile Function‐5 [IIEF‐5]) and female (Female Sexual Function Index [FSFI]) sexual function in relation to age. It can be observed that in both males and females, as age increases, sexual function worsens significantly (*p < 0.05). Data are expressed as median and relative range

A summary of sexual function scores as assessed by FSFI and IIEF‐5 scores, according to BMI class and age, is reported in Figures 1 and 2.

Regarding the results of multivariate logistic regression analysis in the male group, we observed that neither age (*p* = 0.474; OR 1.259, 95% CI 0.670–2.365), age at diagnosis (*p* = 0.658; OR 0.865, 95% CI 0.455–1.644), number of months on a gluten‐free diet (*p* = 0.541; OR 0.981, 95% CI 0.924–1.043), the presence of hypertension (*p* = 0.713; OR 1.661, 95% CI 0.111–2.478), diabetes mellitus (*p* = 0.853; OR 1.184, 95% CI 0.197–7.104) nor BMI (*p* = 0.609; OR 1.068, 95% CI 0.830–1.373) were sexual disease predictors.

We also assessed whether the serum level of anti‐transglutaminase antibodies had any influence on IIEF‐5 score and found no correlation (data not shown).

## 4 DISCUSSION

This cross‐selection observational study demonstrates that SD have a high incidence in CD patients. Half of our female patients reported an FSFI score suggestive for SD and complained of some kind of symptom related to their sexual life, including decreased desire, altered arousal or lubrication, difficulty in attaining orgasm, or some degree of discomfort at intercourse. In the male population, almost 70% of patients did not report any SD. The remaining 30% had some degree of ED that was mild to moderate in the majority of cases.

A normal sexual function is widely recognized as an important determinant of quality of life. This is often impaired in both males and females, with prevalence in the general population of 40%–50% in women independent of age and of 1%–10% in men younger than 40 years, 2%–15% in those from 40 to 49 years, and 20%–40% in those between 60 and 69 years and 43%, respectively.^1^, ^15^, ^16^ SD are defined (according to the DSM‐V) into four major categories (overall, disorders of sexual desire, arousal, orgasm and sexual pain).^3^ In males, the most prevalent disorder is related to ED followed by premature ejaculation, whereas in women dyspareunia, that is, pain during sexual intercourse, and hypoactive desire represents the most common sexual disorders.^4^, ^5^ The etiopathogenesis of SD is widely multifactorial and includes physiological, anatomical, and socio‐cultural differences.^6^ It is well‐known that several immune‐mediated diseases could further affect sexual activity, both directly and indirectly, acting via medications and on body‐image perceptions and desires.^15^, ^16^ Mounting evidence indicates that a number of chronic diseases may be associated with some degree of SD.^17^ A number of patients with chronic gastrointestinal and hepatic disorders have been demonstrated to suffer from SD with a significant alteration of their quality of life.^2^ The pathogenesis of SD in chronic gastrointestinal disorders is multifactorial and may be contributed by psychological problems, chronic inflammation, endothelial dysfunction, endocrine disturbance, and drug related.

In this regard, CD is an immunologically mediated inflammatory disorder of the small bowel common enteropathy which may be associated to a number of extraintestinal manifestations involving different organs.^2^ SD in CD patients might be contributed to by specific nutritional deficiencies because of the malabsorption and/or endocrine dysfunction such as androgen resistance or hyperprolactinemia.^18^ Indeed, in untreated CD patients, the lack of conversion of testosterone to dihydrotestosterone has been demonstrated, because of a deficiency of the peripheral 5a‐reductase. Moreover, CD patients have been reported to have SD and hypogonadism.^19^, ^20^ This suggests that patients with CD should be investigated for any problem related to sexual activity in order to establish a prompt diagnosis and treatment.

Ciacci et al.^8^ showed that untreated CD patients had a significantly lower frequency of intercourse and an overall lower satisfaction regarding their sexual life, which drastically improved after 1 year of gluten‐free diet. Our study in part confirms the findings by Ciacci et al. who, in a small cohort of CD patients (i.e., 55), found a decreased frequency of intercourse and a decreased prevalence of individuals satisfied with their sexual life compared with control, which improved when gluten‐free diet was prescribed. More recently, a study by Zingone et al.^21^ explored the prevalence of hedonistic feelings/anhedonia and sexual pleasure in a cohort of 178 CD patients, reporting any difference in anhedonia or sexual pleasure between CD patients and controls. However, the assessment of SD was not the primary endpoint of the study and the questionnaire used (the Snaith‐Hamilton Pleasure Scale) was not properly designed to explore the sexual life of patients involved, as it includes domains including pleasure regarding other activities (such as hobbies, interactions with other people).^22^

Similarly, the use of drugs may affect sexual function, especially antihypertensive and psychotropic drugs.^23^ In our study, we found that in women, iron‐deficiency anemia and Hashimoto thyroiditis were associated with a low FSFI score. Also, an altered BMI (i.e., lower or higher than normal) was significantly associated with FSFI score. However, it should be considered that in our female population only 23 patients had a BMI >24.99, that is, higher than normal, and only four had a BMI compatible with true obesity. Several evidence shows how BMI within normal limits is associated with better sexual function than obesity or excessive thinness.^24^, ^25^ Additionally, Wekker et al.^26^ showed that improving lifestyle in patients with obesity and infertility was associated, within 6 months, with increased sexual intercourse, improved vaginal lubrication as well as improved overall sexual function. On the other side, use of antianxiety or antidepressant drugs did not seem to be associated with altered sexual function in our female CD population.

In addition, as expressed in Section 3, we observed particularly low FSFI scores in the female population with no sexual partners. While this questionnaire should be prioritized toward a population that had or has sexual activity, it is also true that, given the non‐negligible prevalence of SD that we observed in our CD population, CD could probably represent a barrier in the search for a sexual partner.

In the male CD population, we did not find any correlation with comorbidities. The only variables that were significantly associated to some degree of ED or worsened pleasure scores were bloating, self‐employment, and active or previous smoking. Similarly, increasing age, early age at diagnosis, and high BMI significantly correlated with altered sexual function in male CD patients, even though, at multivariate analysis, these variables were not predictors of SD.

The present study presents different limitations. Firstly, because of its non‐longitudinal nature, we do not have any data regarding SD prior to or after starting a gluten‐free diet. Second, we did not have enough data to correlate SD to the degree of intestinal damage, as assessed by the Marsh‐Oberhuber and Corazza‐Villanacci scores.^27^ Therefore, we cannot draw any conclusion whether the degree of intestinal damage at the diagnosis had a significant influence on the degree of SD.

Finally, our gender assessment did not fully explore sexual preference as well as the possible presence of nonbinary gender patients.

## 5 CONCLUSION

Our study demonstrates that the use of validated questionnaires may be useful for the assessment of sexual dysfunction in celiac disease patients. In this clinical setting, both female and male celiac disease patients show some degree of sexual dysfunction and this was more prevalent in females. Scores evaluating sexual pleasure, arousal, and dyspareunia in women and erectile dysfunction in men are the ones that are mainly impaired. Based on this study, we postulate that evaluation of sexual function through validated questionnaires should be part of the initial clinical assessment in celiac disease patients, in order to promptly identify symptoms of sexual dysfunction and start an early and appropriate therapeutic intervention.

## CONFLICT OF INTEREST

The authors declare no conflict of interest.

## FUNDING INFORMATION

The authors did not receive any funding for this work.

## AUTHOR CONTRIBUTIONS

*Conceptualization*: Marco Romano and Felice Crocetto. *Methodology*: Raffaele Pellegrino and Marco Romano. *Validation*: Carmine Sciorio, Luigi Napolitano, and Biagio Barone. *Formal analysis*: Raffaele Pellegrino. *Investigation*: Lorenzo Romano and Raffaele Pellegrino. *Resources*: Antonietta Gerarda Gravina, Antonio Santonastaso, Caterina Mucherino, and Silvia Astretto. *Data curation*: Antonietta Gerarda Gravina, Antonio Santonastaso, Caterina Mucherino, Silvia Astretto, Luigi Napolitano, Achille Aveta, Savio Domenico Pandolfo, Davide Loizzo, and Francesco Del Giudice. *Writing—original draft*: Lorenzo Romano and Raffaele Pellegrino. *Writing—review and editing*: Biagio Barone and Savio Domenico Pandolfo. *Visualization*: Carmine Sciorio, Biagio Barone, and Matteo Ferro. *Supervision*: Matteo Ferro, Ciro Imbimbo, and Marco Romano. *Project administration*: Marco Romano and Felice Crocetto.
